# Hemorrhagic Shock Due to Traumatic Anterior Cerebral Artery Aneurysm: A Case Report

**DOI:** 10.7759/cureus.39801

**Published:** 2023-05-31

**Authors:** Toshiro Imamoto, Makoto Sawano, Makoto Murase

**Affiliations:** 1 Department of Emergency Medicine and Critical Care, Saitama Medical Center, Saitama Medical University, Kawagoe, JPN

**Keywords:** case report, pseudoaneurysm, aca, traumatic brain injury, shock

## Abstract

Massive subdural hematomas are known to cause hemorrhagic shock in infants and young children. Traumatic cerebral aneurysms are rare and are often noticed in the subacute phase with disorientation due to the rupture of a pseudoaneurysm. No previous studies appear to have clarified the diagnosis of and therapeutic interventions for traumatic cerebral aneurysms identified from computed tomography (CT) on admission. The present case involved an open skull fracture resulting in hemorrhagic shock due to subcutaneous extravasation from an anterior cerebral artery (ACA) pseudoaneurysm.

A seven-year-old boy was accidentally struck by a car after running out into a road. He had an open fracture of the skull and contrast-enhanced CT of the head showed subcutaneous extravasation from the ACA. The patient developed hemorrhagic shock that resolved following the embolization of the ACA with n-butyl-2-cyanoacrylate. Head trauma can cause hemorrhagic shock in the presence of an open wound due to a skull fracture. Contrast-enhanced CT of the head on admission is useful for diagnosis.

## Introduction

Hemostasis is important for the treatment of major bleeding due to trauma [1]. In particular, the thoracic, abdominal, and pelvic cavities are considered the most common sites of massive bleeding [1]. Hemorrhagic shock may occur with bleeding from atypical locations in infants with trauma, such as when a large hematoma is present [2]. According to the Monro-Kellie doctrine, intracranial pressure causes hemorrhage to be self-limiting [3]. Death from head trauma with cerebrovascular injury is thus more likely to be caused by cerebral herniation following hematoma compressing the brain parenchyma than by exsanguination. Traumatic cerebral aneurysms are rare and are often noticed in the subacute phase with disorientation due to the rupture of the pseudoaneurysm [4].

Few previous papers have reported the presence of hemorrhagic shock, which is primarily caused by bleeding from a cerebral aneurysm. The present case involves an open skull fracture resulting in hemorrhagic shock due to subcutaneous extravasation from the anterior cerebral artery (ACA) pseudoaneurysm.

## Case presentation

**Figure 1:**
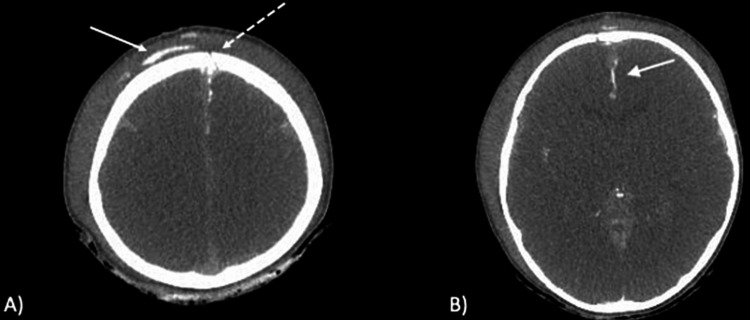
Head CT image shows (A) an extravascular leakage image (white arrow) outside the skull. The extravascular leak is continuous from the intracranial area through the skull fracture (dotted arrow). (B) extravascular leak (white arrow) in the frontal longitudinal of the cerebrum, suggesting that the cause of the hemorrhage may be the ACA.

A seven-year-old boy was accidentally struck by a car after running out into a road. He was unconscious on arrival at the previous hospital, with a Glasgow Coma Scale (GCS) of 6 (E1V1M4; no eye-opening, no verbal response, withdrawal in response to pain), pupil diameter of 5 mm, and bilateral absence of light reflexes. Body weight was 25 kg, blood pressure was 133/90 mmHg, heart rate was 113 beats/min, and respiratory rate was 30 breaths/min with an oxygen saturation of 94% on ambient air. There was significant trauma to the face and persistent bleeding from the oral and nasal cavities. The oral bleeding was followed by fractured teeth and oozing from the nasal cavity. The patient was intubated to secure the airway, but no specific hemostatic measures were taken, including compression hemostasis for external bleeding as a circulatory abnormality. The patient's level of consciousness was less than 8 GCS points, and severe head trauma was suspected. The wound was sutured with a skin stapler, including subcutaneous tissue, but arterial bleeding continued from the wound gap, and the overlying gauze quickly became stained with blood. The patient underwent endotracheal intubation and ventilator management, and computed tomography (CT) of the head without intravenous (IV) contrast and of the chest, abdomen, and pelvis with IV contrast showed fractures of the skull crown and skull base and diffuse cerebral edema. CT showed no obvious damage to the trunk. The initial facility lacked the resources and capability to treat and manage the patient, so transfer to a regional trauma center was arranged after diagnosis. At the initial facility, blood pressure dropped to 44/19 mmHg, leading to cardiopulmonary arrest. After the return of spontaneous circulation following cardiopulmonary resuscitation with blood transfusion, the patient required continuous noradrenaline administration, and he arrived at the regional trauma center in a persistent state of shock with a blood pressure of 70/44 mmHg. Transfer required the patient to be in an ambulance for 15 minutes. Direct compression hemostasis for persistent oral and nasal bleeding was performed. A nasal tampon was placed in the nasal cavity and oral bleeding from a fractured tooth was stopped with bone wax. However, the open wound on the head continued to bleed, with blood dripping from the head onto the floor of the CT room. Blood pressure at this point was 81/43 mmHg with noradrenaline continuously administered at 0.6 μg/kg/min, and the patient remained in a state of shock. Whole-body contrast-enhanced CT (including the head) was repeated. CT angiography (CTA) was not performed. Extravascular leakage was identified in the frontal longitudinal fissure of the cerebrum, and continuous extravascular leakage in the delayed phase was seen from intracranially to the subcutaneous region of the frontal head (Figures 1A, 1B).

**Figure 2:**
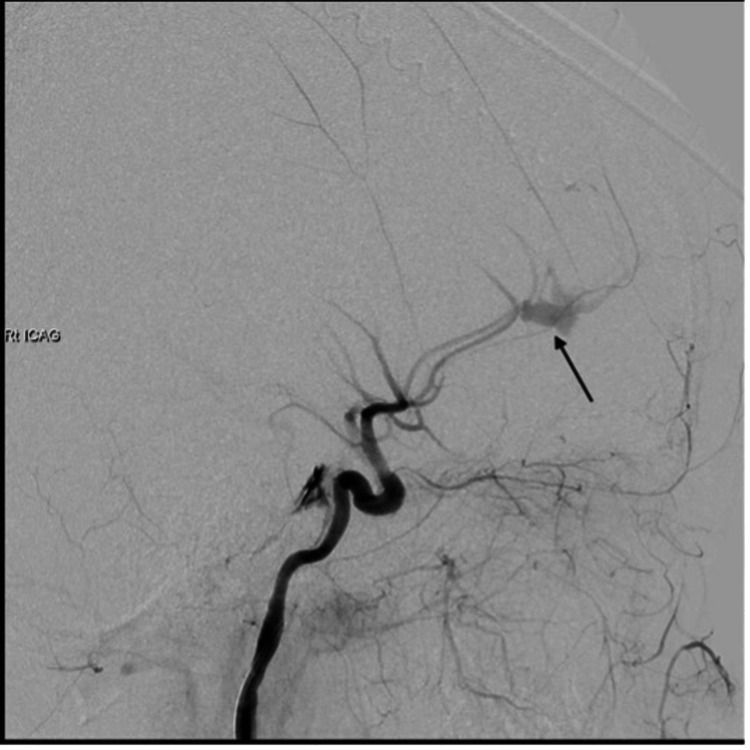
Angiography from common carotid artery shows an open extravascular leak with pseudoaneurysm in the A3 segment of the right ACA (black arrow).

Significant cerebral edema was apparent, and the neurological prognosis was considered poor, but it was considered that hemostasis would save the life of the patient. Blood gas analysis showed acidosis (pH 7.17; reference range: 7.35-7.45). Serum lactate was 6 mmol/L (reference range: <2 mmol/L) and plasma Clauss fibrinogen assay was 1.6 g/L (reference range: 1.5-4.0 g/L), close to the lower limit of normal despite administration of fibrinogen at the previous hospital. The International normalized ratio (PT-INR) was 2.78 (reference range: 0.8-1.2), platelet count was 20,000/μL (reference range: 150,000-400,000/mL), and Hemoglobin was 5.6 g/dL (reference range: 11.5-14.4 g/dL). Following the massive transfusion protocol of the hospital with the diagnosis of disseminated intravascular coagulation, 14 units of red blood cells, 12 units of fresh frozen plasma, 10 units of platelet concentrate, 4 g of fibrinogen concentrate, and 15 units/kg of factor XIII were administered. Blood tests after transcatheter arterial embolization (TAE) showed improvements to PT-INR, 1.21; Clauss fibrinogen, 2.3 g/L; and Hb, 11 g/dL. Thromboelastography was not used, but coagulation markers were checked every hour. Angiography showed an open extravascular leak with pseudoaneurysm in the A3 segment of the right ACA (Figure 2).

**Figure 3:**
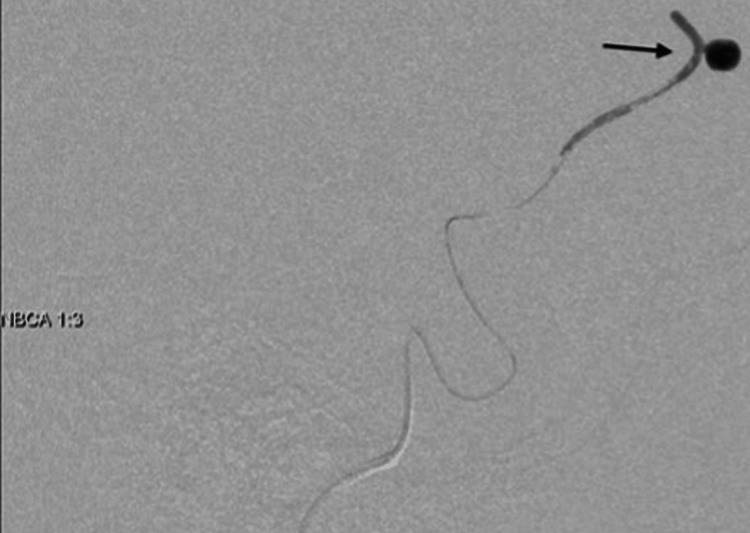
Transcatheter arterial embolization (TAE) performed with NBCA in the area from distally to the A2 segment of the ACA (black arrow).

TAE was therefore performed with n-butyl-2-cyanoacrylate (NBCA)-Lipiodol (NBCA: Lipiodol = 1:3) in the area from distal to the A2 segment of the ACA (Figure 3).

After hemostasis was achieved, the patient was admitted to the intensive care unit. On day 6 after admission, vasopressive agents were no longer required. On day 14 after admission, EEG was flat, and the patient was determined to be clinically brain dead. Discussions were held with his family about whether he should be a donor for organ transplantation, shifted to palliative treatment, or aim for home care with ventilator management and tube feeding after tracheotomy, which remains an option in Japan. The decision was made to pursue home care and he is still in the pediatric ward for a long stay.

## Discussion

Traumatic hemorrhagic shock is a condition that results from trauma leading to hemorrhagic shock. Three main spaces in the body can cause hemorrhagic shock: the thoracic cavity; the abdominal cavity; and the pelvic cavity (as the retroperitoneal space) [1]. The thoracic and abdominal cavities are free spaces in which blood can rapidly accumulate and excessive bleeding can easily cause exsanguination. Injuries to the heart, lungs, and intercostal arteries are the primary causes of intrathoracic hemorrhage, while injuries to the liver, spleen, and mesenteric artery that result in capsular failure represent the primary causes of intraperitoneal hemorrhage. The pelvic cavity is closely associated with retroperitoneal hemorrhage. The retroperitoneal space is a loose space in which hemorrhage spreads more slowly than in free space, but more easily than in tight spaces such as the intramuscular or intraparenchymal spaces in young patients [1]. Multiple traumas involve a combination of these types of hemorrhage, and treatment strategies thus vary from case to case. Of note, in the elderly, subcutaneous hemorrhage that would normally stop spontaneously in a younger patient or bleeding in a tight space can also lead to shock.

Head trauma with injury to the intracranial vasculature usually results in hemorrhage in a closed space. Death often results from respiratory or cardiac arrest due to herniation of the brain as the hematoma compresses the cerebral parenchyma. Hemorrhagic death due to head trauma is rare, but we encountered a case of traumatic hemorrhagic shock caused by ACA injury, in which a pseudoaneurysm and extravascular leakage from the ACA due to open skull fracture led to the same condition as bleeding into free space. We were able to determine that the patient had ACA injury because whole-body multislice (pan-scan) enhanced CT was performed under a hospital protocol, unlike routine imaging from the parietal region. This protocol allowed us to identify the source of the bleeding. We also discuss the benefits and risks of contrast-enhanced imaging of the head in trauma CT imaging.

The identification of extravasation on CTA for endogenous intracerebral hemorrhage has been reported as useful in many cases as a spot sign. On the other hand, few reports have examined the utility of CTA for traumatic intracranial hemorrhage [5]. Letourneau et al. reported that in a study of 60 cases with traumatic intracranial hemorrhage (intracranial hemorrhage, subdural hematoma, or epidural hematoma), extravasation was found in 30 cases (50%). This correlated with increased hematoma and poor outcomes compared to cases with no extravasation [6]. Based on such reports, we believe that the evaluation of images showing extravascular leakage for traumatic intracranial hemorrhage is useful in cases of severe head trauma and multiple trauma.

Given the principles underlying contrast-enhanced CT, three possible disadvantages can be incurred from using contrast-enhanced CT that includes the head. First, the brainstem region may be obscured. Second, CT attenuation values may be insufficient to allow the construction of a detailed three-dimensional CTA model. Third, CT attenuation values of the contrast medium are too high from the injection site to the aortic arch, making an evaluation of bleeding in the same area slightly more difficult. Since the quality of CT alone is sufficient to determine the presence of intracranial extravascular leakage in cases of severe trauma, this is unlikely to represent a significant disadvantage. The Japanese guidelines for initial trauma care define the imaging range for whole-body CT as extending from the skull base to the pelvis in the arterial phase and from the chest to the pelvis in the venous phase. This is the same as the Advanced Trauma Life Support (ATLS) in the United States [7]. Although there is no mention of imaging up to the parietal region at the guideline level, we consider the following. We believe that whole-body CT including the head is useful for an initial evaluation in cases of trauma where complications of head trauma are anticipated and in cases of severe trauma where multiple injuries will require surgery or where interventional radiology and follow-up CT of the head within a short period of time will not be feasible. This protocol is useful for predicting hematoma growth. The imaging protocol for cases of severe multiple trauma with impaired consciousness should include the head within the area for routine contrast-enhanced CT.

The fact that the brainstem may be somewhat opaque is not disadvantageous if CT of the head without IV contrast is performed before pan-scan CT. Imaging all trauma cases as a routine should be avoided from the perspective of limiting radiation exposure. In our institution, we perform CT of the head as CTA rather than contrast-enhanced CT of the whole body when the findings from CT without IV contrast indicate that CTA is warranted. CTA and contrast-enhanced CT including the head on whole-body CT are different and should be clearly distinguished.

## Conclusions

In this case, the patient presented with hemorrhagic shock due to bleeding from a traumatic intracranial pseudoaneurysm associated with an open skull fracture. Contrast-enhanced CT of the whole body including the head enabled diagnosis immediately after the injury and avoided hemorrhagic death. A traumatic intracranial pseudoaneurysm can also cause hemorrhagic shock if the skull is open. In whole-body CT of trauma, contrast-enhanced CT is often performed for the trunk and CT without contrast for the head, and intracranial traumatic pseudoaneurysms are easily overlooked on initial CT. These two points should be kept in mind by emergency physicians.
